# Navigating pathways to automated personality prediction: a comparative study of small and medium language models

**DOI:** 10.3389/fdata.2024.1387325

**Published:** 2024-09-13

**Authors:** Fatima Habib, Zeeshan Ali, Akbar Azam, Komal Kamran, Fahad Mansoor Pasha

**Affiliations:** ^1^FAST School of Management, National University of Computer and Emerging Sciences, Lahore, Pakistan; ^2^Oxford Brookes Business School, Oxford Brookes University, Oxford, United Kingdom; ^3^Faculty of Business Administration, Lahore School of Economics, Lahore, Pakistan

**Keywords:** automated personality prediction, Big Five personality model, natural language processing, social media text, muti-output regression, large language models, psychometric analysis

## Abstract

**Introduction:**

Recent advancements in Natural Language Processing (NLP) and widely available social media data have made it possible to predict human personalities in various computational applications. In this context, pre-trained Large Language Models (LLMs) have gained recognition for their exceptional performance in NLP benchmarks. However, these models require substantial computational resources, escalating their carbon and water footprint. Consequently, a shift toward more computationally efficient smaller models is observed.

**Methods:**

This study compares a small model ALBERT (11.8M parameters) with a larger model, RoBERTa (125M parameters) in predicting big five personality traits. It utilizes the PANDORA dataset comprising Reddit comments, processing them on a Tesla P100-PCIE-16GB GPU. The study customized both models to support multi-output regression and added two linear layers for fine-grained regression analysis.

**Results:**

Results are evaluated on Mean Squared Error (MSE) and Root Mean Squared Error (RMSE), considering the computational resources consumed during training. While ALBERT consumed lower levels of system memory with lower heat emission, it took higher computation time compared to RoBERTa. The study produced comparable levels of MSE, RMSE, and training loss reduction.

**Discussion:**

This highlights the influence of training data quality on the model's performance, outweighing the significance of model size. Theoretical and practical implications are also discussed.

## 1 Introduction

Human personality, a multifaceted account of behavior and traits, has been a fascinating subject for psychology researchers for several decades. Human personality acts as a driving force for steering behavior, emotional responses, and social interactions (Youyou et al., 2015). Digital records of human behavior have been empirically proven to offer effective personality assessments (Quercia et al., 2011; Kosinski et al., 2013), directly correlating with better decision-making (Letzring and Human, 2014). Various studies have highlighted the potential of Natural Language Processing (NLP) (Christian et al., 2021; Berggren et al., 2024) and empirically predicted personality traits using Machine Learning (ML) (Tadesse et al., 2018) and deep learning models (Tandera et al., 2017; Yu and Markov, 2017). With ongoing advancements in computation, Large Language Models (LLMs) have experienced a significant surge in popularity. Pre-trained LLMs outperform state-of-the-art models in downstream NLP tasks, particularly personality prediction (Kazameini et al., 2020; Theil et al., 2023). Several studies indicate that Automated Personality Prediction (APP) with LLMs can significantly improve the accuracy and responsiveness of computing decisions, resulting in valuable insights (Jukić et al., 2022; Matz et al., 2023; Peters and Matz, 2023).

Contemporary technologies with big data have significantly improved automated predictions of personality (Ihsan and Furnham, 2018). This development continues to reshape the societal processes across diverse domains including social media, online education, business functions, and the electoral process (Alexander et al., 2020). Various researchers have executed APP in diverse contexts from the study of CEO risk-taking personalities, personality-job fit, and brand-follower personality matches to music recommendation systems tailored to personality (Wynekoop and Walz, 2000; Tang et al., 2018; Tomat et al., 2021; Kleć et al., 2023; Theil et al., 2023).

Traditionally, personality has been analyzed using a personality inventory of self-report questionnaires (Zhong et al., 2018). Amidst all potential sources of personality data, social media platforms stand out the most, given the frequency of their use by immensely large and varying demographics (Alexander et al., 2020). In behavioral sciences, self-report scales are often affected by social desirability bias as people tend to give socially acceptable responses that are different from their true feelings (Podsakoff et al., 2003). Conversely, on social media, users exhibit more naturalistic behavior as they are not being observed (Funder, 2012), resulting in a potentially better reflection of their personality. Since the digital footprint allows data collection of a larger population in real-time, individual as well as group-level dynamics can be better understood (Golder and Macy, 2014). In this era of rapid computational advancement, APP with its hidden intricacies, has arisen as a promising frontier in learning human psychology. To support APP, the linguistic information from our everyday language can help us effectively draw inferences about a person's personality (Kulkarni et al., 2018). Scholars have proposed various theoretical underpinnings to explain complex human personality. Among various models, the Big Five model is widely used as a robust and meticulous framework (Poropat, 2009). Table 1 exhibits the Big Five traits, their corresponding social aspects, and specific words used (Yarkoni, 2010; López-Pabón and Orozco-Arroyave, 2022).

**Table 1:** Big Five traits with corresponding social aspects and frequently used words.

**Big Five trait**	**Social aspect explained**	**Frequently used words**
Conscientiousness	Careless vs. self-disciplined	Completed, adventure, boring
Openness to experience	Dull vs. intellectual	Folk, humans, art, poetry, culture
Agreeableness	Uncooperative vs. obliging	Wonderful, together, felt, morning
Extraversion	Shy vs. friendly	Restaurant, dancing, shots, bar
Neuroticism	Emotionally unstable vs. calm	Awful, lazy, worse, irony, depressing

Various statistical and Machine Learning (ML) methods have been developed and tested, producing APP knowledge. These include Support Vector Machines (SVM) (Berggren et al., 2024), Naïve Bayes (Cui and Qi, 2017), and booster classifiers (Tadesse et al., 2018). Deep learning models including Convolutional Neural Networks (CNN), Long Short-Term Memory (LSTM), and Recurrent Neural Networks (RNN) have also been investigated for predicting personality through NLP (Majumder et al., 2017; Tandera et al., 2017; Yu and Markov, 2017; Deilami et al., 2022). With promising improvements in newer technologies, research on APP is continuing to gain momentum.

In the present-day context, there has been a dramatic increase in transformers-based LLM-related investigations (Kumar and Renuka, 2023). While text analytics has gained considerable attention in research, it can be refined further with fine-tuning trials using LLMs demonstrating state-of-the-art performance (Kjell et al., 2023). This refinement enhances real-world applications, particularly downstream NLP tasks such as text classification and sentiment analysis due to the capability of LLMs to focus on the context of words (Lewis et al., 2020; Kjell et al., 2023). Transformers (Vaswani et al., 2017) outperform previous techniques in text analysis due to the self-attention mechanism for capturing long-distance dependencies; parallel training for faster computation; and versatile capabilities facilitated by their unique architecture. Various studies have experimented with diverse models, such as BERT (Devlin et al., 2019; Zhao and Wong, 2023), RoBERTa (Putra and Setiawan, 2022), ULMFiT (El-Demerdash et al., 2021), and DistilBERT (Sanh et al., 2019), to harness the full potential of transformers (Rajapaksha et al., 2021).

Despite the sheer rise in LLMs, their deployment poses a few major challenges. One of the primary obstacles is the ever-increasing consumption of scarce resources, as these models are large and data-intensive (Schick and Schütze, 2020; Fu et al., 2023; Hsieh et al., 2023). These sizeable models extensively consume computational and memory resources (Hsieh et al., 2023). Thereby, contributing to energy inefficiency and a large carbon footprint through carbon dioxide equivalent emissions (CO_2_e) into the environment (Schick and Schütze, 2020; Patterson et al., 2021). Comparative estimates of CO_2_e exhibit a significant difference between the T5 model (11 billion parameters) and the GPT-3 (175 billion parameters), with 46.7 and 552.1 metric tons of CO_2_e, respectively (Patterson et al., 2021). Additionally, LLMs are also associated with the substantial water footprint crisis, an often-overlooked environmental threat (Li et al., 2023; Rillig et al., 2023). Conversely, smaller language models offer an opportunity to mitigate the risks accompanying LLMs. These models can be fine-tuned to be computationally efficient, match, or sometimes even surpass the accuracy of larger models (Kazameini et al., 2020).

Previous studies have predominantly emphasized increasing LLM size as a means to increase accuracy. Nevertheless, ongoing research is centered on attaining higher accuracy with smaller models. This study focuses on fine-tuning a small language model (Albert-Base 2 with 11.8 million parameters) in comparison with a larger one (Roberta-Base with 125 million parameters) for APP. By comparing these two language models for multi-output personality prediction, this research makes the following contributions to the existing literature on APP. First, the comparative analysis provides insights into the optimal selection of a language model offering a comparative level of error reduction for APP. Second, the results offer perspectives on minimizing computational resource constraints encompassing time consumption, heat emission, and computational power usage. Third, it examines the viability of predicting personality using a continuous scale for each of the five traits of the Big Five Model, through multi-output regression. As recommended in several researches (Feizi-Derakhshi et al., 2022; Johnson and Murty, 2023).

## 2 Background and literature

“The web sees everything and forgets nothing” (Golder and Macy, 2014). The digital footprint has enabled the field of computational social science to extend its mining into human behavior on a massive scale. This big data analysis has led to the fine-grained investigation of critical phenomena such as social network analysis (Letzring and Human, 2014), public opinion (Christian et al., 2021; Berggren et al., 2024), and social influence on political mobilization during electoral events (Cui and Qi, 2017; Tandera et al., 2017; Tadesse et al., 2018). Moreover, time spent on social media can help decipher the emotional states of smartphone users' indicating boredom and loneliness (Kazameini et al., 2020; Theil et al., 2023). In turn, such emotional states and tones can help recognize user demographic traits (Volkova and Bachrach, 2016). While social influence can be assessed from the friends on a social media network, the use of language can be indicative of user intention leading to the detection of depression and suicidal tendencies (Jukić et al., 2022; Matz et al., 2023; Peters and Matz, 2023). Social media data, when utilized responsibly, carries immense potential to significantly benefit the community though data-driven decision-making.

**Figure 1:**
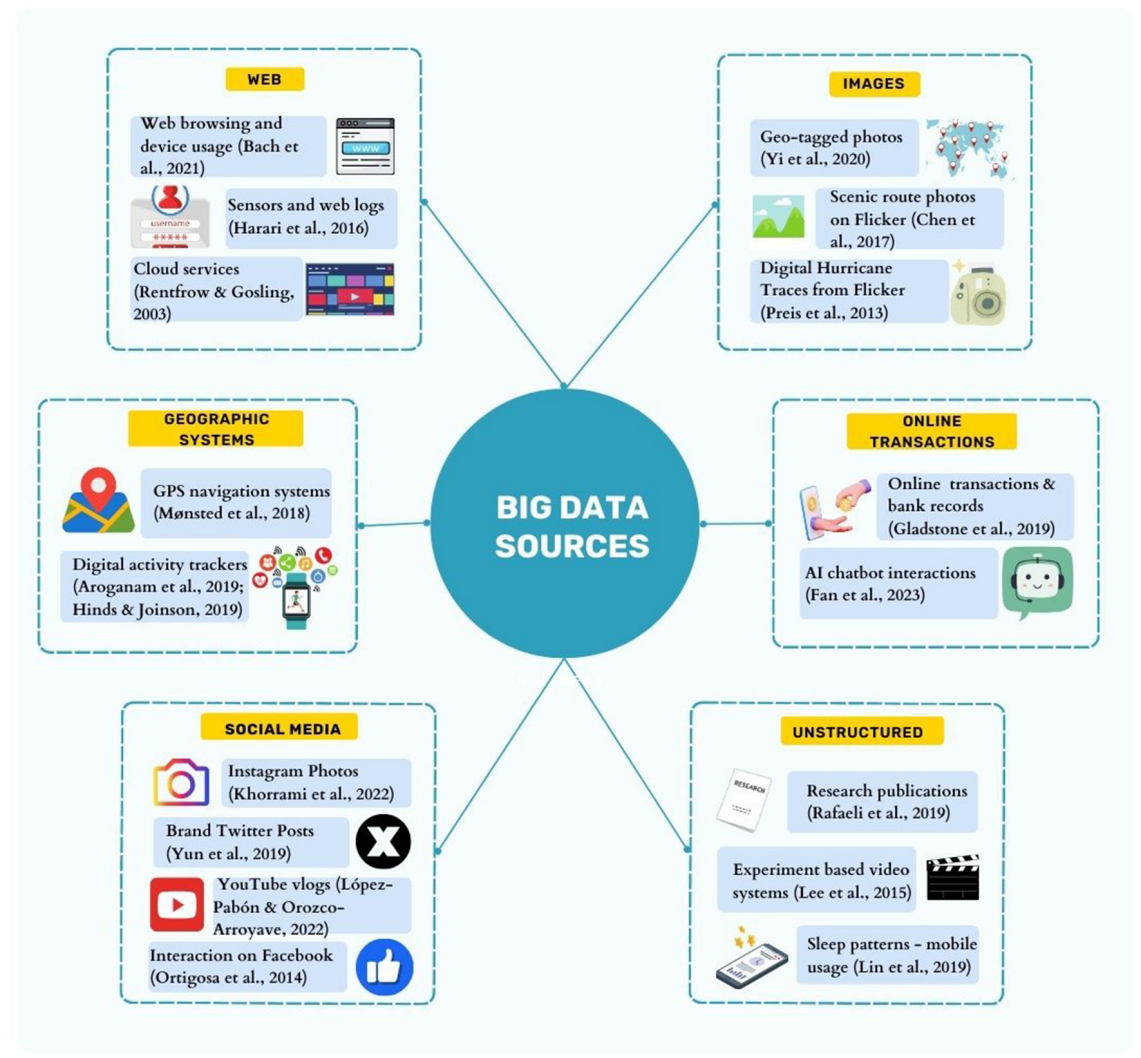
Diverse sources of Big Data for model training and evaluation.

Pivoting to the business sphere, social media has acted as a catalyst for fostering analytical insights across diverse business functions. Consumer sentiments and attitudes toward culturally diverse brands can be inferred from the data available on social networks and blogs (Alexander et al., 2020). Such data can offer valuable insights into user interests across a diverse spectrum of health, religion, movies, music, and arts (Lewenberg et al., 2015). Emotional drivers and user influence on social media can be assessed and integrated into information systems for enhanced business decision-making (Chung and Zeng, 2020). Data harnessed from social media discourse can also help us infer demographic information such as income, gender, opinions, sentiments, and personality traits (Wynekoop and Walz, 2000; Volkova et al., 2016; Hinds and Joinson, 2018; Tang et al., 2018; Tomat et al., 2021; Kleć et al., 2023; Theil et al., 2023). Such pertinent insights from digital traces (as exhibited in Figure 1) can be extremely beneficial when implemented in targeted advertising, marketing, and customer relationships (Kosinski et al., 2014; Matz et al., 2017). While digital footprints can be utilized in these diverse applications, this paper primarily focuses on their use in APP.

### 2.1 Automated personality prediction

Personality has been a subject of extensive research, which has significantly contributed to our understanding of human behavior and actions (Putra and Setiawan, 2022). Funder (2012) describes personality as patterns of emotions, thoughts, and behavior consistent across situations and over time. Personality distinguishes individuals and forms clusters of individuals to reveal consistent behavioral patterns (Kulkarni et al., 2018). It refers to the characteristic amalgamations of emotional reactions developed from biological makeup and circumstantial factors, resulting in consistent differences (Karanatsiou et al., 2022).

Numerous studies have investigated the diverse aspects of human personality. Among various others such as the Personality Enneagrams (Sutton et al., 2013) and Myers Briggs Type Indicator (MBTI) (Gjurković and Šnajder, 2018), the Big Five model is appreciated as a widely used framework for psychological assessment (Zimmermann et al., 2019; Gjurkovic et al., 2021). It provides scores for five OCEAN (Openness, Conscientiousness, Extraversion, Agreeableness, Neuroticism) traits that rate the respondents on a continuum (McCrae and John, 1992). The Big Five personality model is deemed one of the most credible and widely known scales for gauging personality against five traits of natural language for fine-grained analysis (Goldberg, 1982; Phan and Rauthmann, 2021). In the new technologically advanced global economy, the upsurge in digital data has posed immense opportunities for data utilization. Digital footprint entails several means of assessing personality. Personality traits can be expressed through likes, comments, musical preferences (Nave et al., 2018), pictures (Segalin et al., 2017), product selection (Hirsh et al., 2012), text, and language (Tutaysalgir et al., 2019; Mehta et al., 2020a). Together, these studies indicate the pertinent role of digital data in personality detection.

### 2.2 Personality and prediction models

Early research on APP focused on traditional statistical methods. Tausczik and Pennebaker (2010) used Linguistic Inquiry and Word Count (LIWC) to perform a psychometric analysis of words. Psycholinguistic feature extraction formed the foundation for Decision Trees, SVM, Naïve Bayes, and several other traditional machine-learning classifiers for personality identification analyzing text (Quercia et al., 2011; Alam et al., 2013; Markovikj et al., 2013; Mohammad and Kiritchenko, 2013). Later, ensemble models, basic neural networks, and text-mining experiments elevated the sphere of APP (Peng et al., 2015; Cui and Qi, 2017; Tadesse et al., 2018; Yang and Huang, 2019). Taken together, these studies highlight an important theme of personality identification, opening doors to the continuous evolution of methodology. In essence, the literature largely focuses on personality detection employing classification methods, with limited attention given to personality prediction as a multi-output regression problem.

Literature has extensively explored the construct of personality prediction from social media texts. Deep learning methods are expected to yield superior results owing to their automatic feature extraction capabilities, unique structures, noteworthy performance, and relatively low computational cost (Xue et al., 2018; Deilami et al., 2022). It is worth mentioning that, in recent years, notable deep neural networks exemplifying personality prediction have emerged. The neural networks employed in APP encompass embeddings, Convolutional Neural Networks, Recurrent Neural Networks, and Long Short-Term Memory Networks with various experiments in combinations (Majumder et al., 2017; Tandera et al., 2017; Yu and Markov, 2017; Sun et al., 2018; Xue et al., 2018). Following the escalating momentum in deep learning, Mehta et al. (2020b) reviewed the advances in APP based on deep learning, This review highlighted that the quality of training data is a significant determinant of the strength of ML models.

What follows is a comprehensive account of the latest developments in the field with pre-trained LLMs built on transformers' attention mechanism. Transformers have significant advantages over previous methods of text analysis such as RNNs and CNNs. Their self-attention mechanism captures long-range dependencies and contextual information in the text (Vaswani et al., 2017), while the parallelized architecture leads to faster training and inference times (Fan et al., 2023b). Moreover, the encoder-decoder framework, birectional context, and multihead attention enable transformers to effectively handle diverse NLP tasks (Rothman, 2021). LLM inference requires lower programming and computational resources after being fine-tuned (Church et al., 2021). Since the base models are generic, they are required to be fine-tuned for a specific downstream task at some point. Fine tuning implies the capability to change the layers expressing more control over the output (Church et al., 2021). Prior fine-tuning using various training procedures can help reduce the burden on the computational resources at the inference stage which consumes more resources as compared to the training process (Schick and Schütze, 2020; Strubell et al., 2020). Despite the significance of fine-tuning LLMs, a few researchers such as Tomat et al. (2021) and Wynekoop and Walz (2000) have started exploring the viability of zero-shot learning without explicit training or fine-tuning it for the specific task. However, research on zero-shot learning is still in the nascent stages, as far as its reported response accuracies are concerned.

LLMs exhibit strong transferability facilitating emotion detection outperforming state-of-the-art models (Peng et al., 2024). Empirical investigations on Bard, GPT, and TAS have claimed that LLMs when trained on sufficient data can achieve or even surpass the benchmark of human emotional intelligence (Patel and Fan, 2023). Such progressions have facilitated much complex Emotional Support Conversations which can prominently elevate customer service chats, counseling, psychotherapy, and mental health support (Zhang et al., 2024). GPT 3.5 has been fine-tuned to detect the target demographic aligning itself when used in public-dealing tools showing varying accuracies (Sicilia et al., 2024). Fine-tuning BERT-like LMs on text authored by balanced demographic cohorts can mitigate the biases that usually arise (Garimella et al., 2022). Alpaca has been successfully fine-tuned to predict survey responses escalating opinion mining and trend analysis of diverse social issues (Kim et al., 2023). The proliferation of LLMs has facilitated a wide array of applications in diverse fields. Among these, a large body of research emphasized the automated prediction of personality through LLMs. Using transfer learning, various investigations have been conducted employing attentive networks such as BERT, RoBERTa, and XLNet with varying combinations, evaluated on text datasets including FriendsPersona, Reddit comment, and Facebook (Jiang et al., 2020; Kazameini et al., 2020; Christian et al., 2021; Gjurkovic et al., 2021).

One of the drawbacks observed in previous studies was the concentration on the classification of personality traits into labels. However, this does not accurately reflect the continuous nature of these traits in humans (Mehta et al., 2020a; Feizi-Derakhshi et al., 2022; Johnson and Murty, 2023). By contrast, predicting personality using regression analysis provides a more realistic representation of an individual's traits. Only a few studies have employed regression as a technique to assess Big Five personality traits (Xue et al., 2018; López-Pabón and Orozco-Arroyave, 2022). Following the proposed methods in the aforementioned studies, our research is focused on multi-output regression as a method to assess the Big Five personality traits from the text analysis of Reddit comments.

Additionally, despite all the developments made by APP using various models as shown in Table 2, there are a few challenges in the continuation of research on LLMs and their application in the industry settings. López-Pabón and Orozco-Arroyave (2022) assert that LLMs tend to be computationally expensive, water-intensive, costly, and time-consuming. They also suggest that further research is needed to compare large and small models. A few studies have advocated the importance of smaller and computationally efficient models for real-time inference. By specializing in specific tasks using model specialization, smaller models derived from larger ones can achieve comparable performance, effectively leveraging the capabilities of large models (Araci, 2019; Yang et al., 2020; Fu et al., 2023). In essence, smaller models have been proposed to achieve similar levels of accuracy, sometimes outperforming larger models (Hsieh et al., 2023). They also require less training data, are more computationally efficient, produce less carbon footprint, and are ultimately cost-effective (Schick and Schütze, 2020; Fu et al., 2023). Therefore, the research is shifting toward the viability of smaller language models with comparable performance. In this study, we are aiming to compare a smaller language model with a larger one, in terms of training loss and resource consumption.

**Table 2:** Models used in previous research and their evaluation metrics.

**Research**	**Model**	**C**	**R**	**Evaluation metrics**
**Statistical and machine learning models**				
Quercia et al. (2011)	M5 algorithm		✓	RMSE
Markovikj et al. (2013)	Simple minimal optimization (SMO) and boost algorithms with POS Tag, Afinn and H4Lvd parameters	✓		TP rate, FP rate, precision, recall, ROC Area
Mohammad and Kiritchenko (2013)	SVM	✓		Recall, accuracy and F1-score
Alam et al. (2013)	SMO (sequential minimal optimization for SVM), Bayesian Logistic Regression (BLR) and Multinomial Naïve Bayes (MNB) sparse modeling	✓		Macro-averaged precision, recall and F1; weighted average accuracy (WA) and un-weighted average accuracy (UA)
Peng et al. (2015)	SVM	✓		Precision and recall
Cui and Qi (2017)	Comparison of SVM, NB classifier and a basic NN with ReLU activation function	✓		Accuracy
Tadesse et al. (2018)	XGBoost classifier	✓		Accuracy
Tutaysalgir et al. (2019)	Clustering algorithms	✓		Silhouette coefficient scores
Yang and Huang (2019)	M5 Regression Tree and SVM		✓	MSE
**Deep learning models**				
Majumder et al. (2017)	Word2vec embeddings with CNN layer	✓		Accuracy
Tandera et al. (2017)	MLP (multi-layer perceptron), LSTM, GRU (gated recurrent unit), and CNN 1D (1-dimensional)	✓		Accuracy
Xue et al. (2018)	Hierarchical DNN (AttRCNN) CNN-based inception structure		✓	MAE
Yu and Markov (2017)	Multi-model (FC, CNN with RNN)	✓		F1 and accuracy
Sun et al. (2018)	Multi-model CLSTM (CNN and BiLSTM)	✓		Precision
Deilami et al. (2022)	CNN with AdaBoost	✓		Accuracy and MAE
Quwaider et al. (2023)	Artificial NN	✓		Accuracy
**Pre-trained language models**				
Jiang et al. (2020)	RoBERTa	✓		Accuracy
Keh and Cheng (2019)	Fine-tuned BERT	✓		Accuracy
Kazameini et al. (2020)	BERT with Bagged-SVM classifier	✓		Accuracy
Gjurkovic et al. (2021)	BERT with NN		✓	r between Big 5 and MBTI results
López-Pabón and Orozco-Arroyave (2022)	Word2Vec, GloVe, and BERT (base and large)	✓	✓	Regression (R square, MAE and RMSE) and classification (F1, accuracy and AUC)
Mehta et al. (2020a)	BERT (base and large)	✓		Accuracy
Ramezani et al. (2022)	KGrAt-Net (knowledge graph attention network text classifer)	✓		Recall, precision, accuracy and F1-score
Theil et al. (2023)	BERT-base and RoBERTa-base		✓	MAE
Johnson and Murty (2023)	Enhanced knowledge graph with BERT	✓		Recall, precision, accuracy and F1-score
Matz et al. (2023)	GPT-3		✓	R
Peters and Matz (2023)	GPT-3.5 and GPT 4 with zero-shot learning		✓	R

R, regression; C, classification.

Several recent studies have focused on APP from text and other sources such as images, voice, and video (Kazameini et al., 2020; Moreno et al., 2021). However, it is still at a developing stage in the field of business and artificial intelligence. Previous studies have predominantly emphasized increasing LLM size as a means to increase accuracy. The majority of which have employed classification techniques for personality predictions. Contrary to these researches, we propose a small pre-trained language model ALBERT in comparison with a larger pre-trained language model RoBERTa for personality prediction from the text of Reddit comments. Unlike the classification approach, we also propose multi-output regression to produce continuous prediction scores for five personality traits simultaneously. We evaluate the models based on loss function particularly, error reduction. Additionally, we assess the training process differences given computational GPU resource consumption, commitment, and heat emission. We demonstrate that small language models exhibit performance comparable to ten times large language models in APP owing to the similarity in training data quality.

## 3 Materials and methods

**Figure 2:**
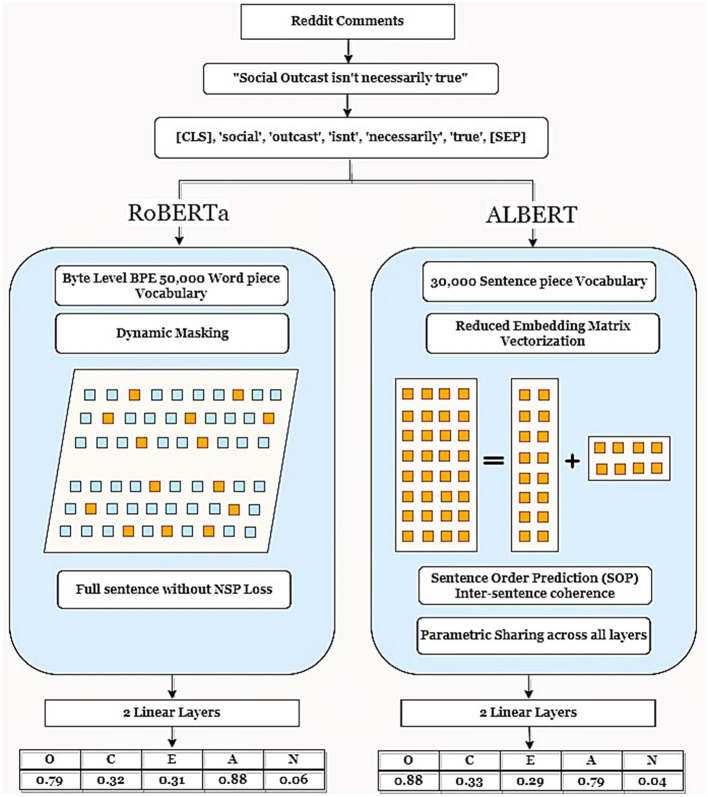
The architecture of RoBERTa and ALBERT with proposed changes and training dataset.

We aim to compare the performance of a large and a small language model by examining the training loss, time-to-train of the pre-trained models, and GPU computation resources consumed while keeping the training parameters constant to train both models.^1^ Figure 2 illustrates the flow chart of our study detailing the flow of data preprocessing, the architectural differences between RoBERTa and ALBERT, and the customization of the model at the last layer.

### 3.1 Dataset

The dataset used for training and fine-tuning this model is titled Personality And Demographics Of Reddit Authors (PANDORA). Gjurković and Šnajder (2018) presented this large-scale dataset collected from the social media platform Reddit.com. Despite being a popular discussion website, it is often overlooked for personality prediction tasks. It encompasses a wide array of topics while preserving user anonymity (Jukić et al., 2022). It started with MBTI categories for 9,000 Reddit users. Later, Gjurkovic et al. (2021) added the Enneagrams and the Big Five scores which resulted in a comprehensive collection of comments posted on reddit.com by 10,288 users. This dataset has been used to support various research focused on predicting personality from text. Li et al. (2021) used it for personality prediction through multi-task learning whereas Jukić et al. (2022) utilized PANDORA to explore the relationship between evaluative language and personality traits. Moreover, Radisavljević et al. (2023) attempted to create a similarity connection among multiple personality models including the Big Five, the Enneagrams, and MBTI.

We selected the Big Five model for our study and extracted author profiles of ~1,608 users, with a total of 27,859 comments from the dataset. The final dataset can be accessed at Habib (2024). Table 3 displays a statistical summary of the original PANDORA Big Five subset while Table 4 presents the statistical account of each of the five traits. Before the training, the dataset was pre-processed to eliminate any noise that could distort the analysis (López-Pabón and Orozco-Arroyave, 2022). Reddit data, which were already anonymous, were further pseudonymized to protect the privacy of the authors, as suggested by Volodina et al. (2020).

**Table 3:** PANDORA statistics summary (Big Five model).

**Property**	**Count**
Number of comments	27,859
Average word count	44.88
Maximum words	3,430
Minimum words	1
Average sentences	2.5
Maximum sentences	71
Minimum sentences	1

**Table 4:** Statistics of personality scores for five traits.

**Property**	**Agreeableness**	**Openness**	**Conscientiousness**	**Extraversion**	**Neuroticism**
Count	27,859.00	27,859.00	27,859.00	27,859.00	27,859.00
Mean	37.213450	67.675365	30.006605	35.726139	49.522722
Std	29.833761	22.208940	27.382423	30.034822	29.106680
Min	0.00	9.00	1.00	0.00	0.00
25%	9.00	50.00	9.00	7.00	20.00
50%	38.00	74.00	20.00	31.00	50.00
75%	57.00	85.00	43.00	59.00	72.00
Max	99.00	98.00	98.00	99.00	99.00

### 3.2 Data pre-processing

The Natural Language Toolkit (NLTK) library of Python (Wang and Hu, 2021) was used to preprocess the text in the dataset.^2^ The text was standardized by converting it into lowercase and removing any hyperlinks and URLs, punctuation, new lines, and special characters. Additionally, the text was tokenized to represent the input text as a sequence of word tokens. Lemmatization was avoided to preserve the linguistic context (Ramezani et al., 2022). Removing stop words has been shown to have no significant impact on the performance of LLMs (Qiao et al., 2019). Therefore, stop words have been retained to maintain the contextual integrity of natural language patterns, essential to LLMs functionality. Afterward, sentences with fewer than five words were filtered out, and non-English comments were removed through NLP's language detection process (Rajanak et al., 2023), using the LangDetect package in Python. The final dataset contained only English sentences to maintain uniformity for better fine-tuning (see Table 5).

**Table 5:** Pre-processed text from the training dataset and corresponding Big Five Scores.

	**Text**	**AGR**	**OPN**	**CON**	**EXT**	**NEU**
1	His name was kim kimble originally wow thats some messed up parents	9	61	13	4	72
2	Theyre better than the normal posts on ryugioh id rather have them then the same topic posted multiple times in the week after the banlist	50	85	50	85	50
3	How the fuck does this even happen hi youre cute you too ive had a crush on you for a while um i uh inserts finger in butthole	15	85	15	85	15
4	It probably does ive learned a lot about myself by browsing this subreddit over the months	71	53	17	3	31
5	Yea those are the same sound to me still	64	44	33	8	88
6	Long term shifting is the cart titans gimmick though the fact that she can do it doesnt mean eren can	50	85	50	85	50
7	Texas is molly weasley i love it	79	84	86	53	1
8	Yeah those are good points my experiences with recruiting is all with really open ended type work that	85	95	15	50	15

AGR, agreeableness; OPN, openness; CON, conscientiousness; EXT, extraversion; NEU, neuroticism.

### 3.3 Model comparison and customization

This paper proposes a comparison of two pre-trained language models, RoBERTa and ALBERT. Each model was customized using two additional linear layers. Liu et al. (2019) introduced RoBERTa using transformers as the underlying mechanism (Kumar and Renuka, 2023). Liu et al. (2019) claim that RoBERTa has been trained on a large English corpus of more than 50,000 byte-level Byte-Pair encoding tokenized vocabulary the masking patterns were dynamically altered, adding to the robustness of the results by eliminating duplicate data during training. RoBERTa's focus is on understanding language. Hence, RoBERTa is deemed to be one of the top-performing models for predicting personality traits (Theil et al., 2023). On the other hand, ALBERT was selected to compare the results of RoBERTa with those of a more modestly sized model. As explained by Lan et al. (2019), ALBERT shares the same architecture as BERT, analogous to the training and fine-tuning processes. It uses matrix-factorized embeddings with sentence-order predictions to better comprehend sentence connections. It carries smaller embedding sizes and also shares parameters across all layers, requiring less memory to store the parameter weights. The cross-layer parameter sharing helps the model to converge faster and enhance parameter efficiency (Plummer et al., 2020). The ALBERT model was selected owing to its smaller size and efficient handling of contextualized text representations.

**Figure 3:**
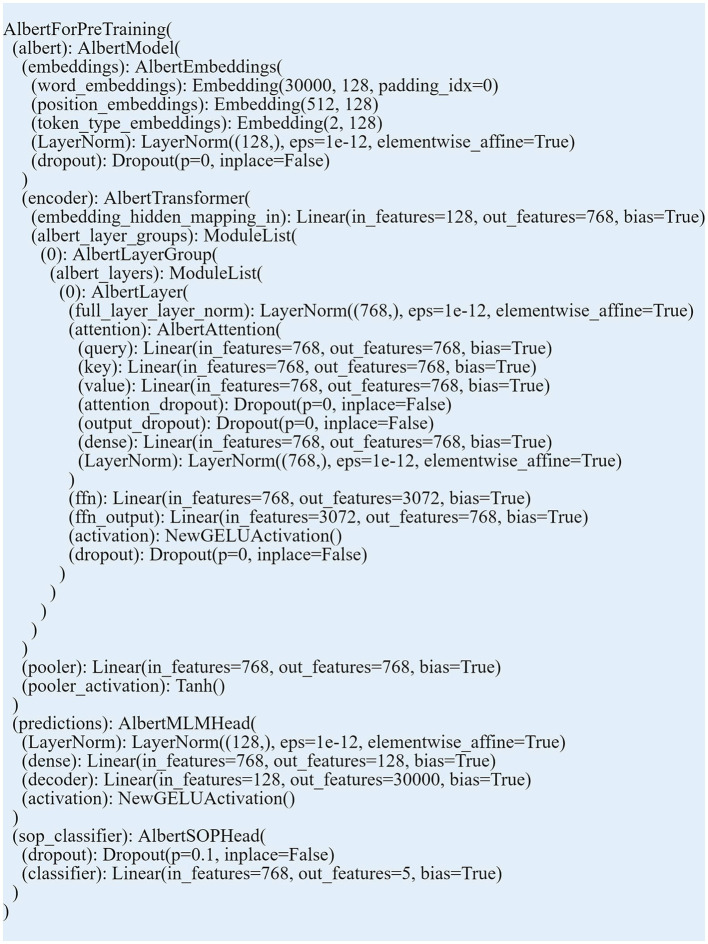
Structure of ALBERT model customized for regression with additional layers.

To accomplish the multi-output regression proposed in this study, both RoBERTa and ALBERT models were customized to handle regression tasks with multiple outputs. ALBERT and RoBERTa models from the Hugging Face Transformers library were leveraged as the core component of the custom models. Pretrained on a large corpus, these models are capable of contextual understanding of language (Devlin et al., 2019). Linear layers were added to each model, followed by an activation function. Initially, the model processes an input text sequence and generates a contextualized representation labeled “the_last_hidden_state.” The mean of this output is computed into “a pooled output” which represents the summary of the input text. This pooled output is then passed through the linear layers added, to produce the final regression output. The hidden and output layers of these models carry the free parameters which can be altered by adding new trainable layers and an output layer. Such customization can be viable for multi-output regression utilizing the transfer learning technique (Emami and Martinez-Munoz, 2024). The first linear layer mapped the hidden size of contextualized word embeddings into a 128-dimensional vector. This transformation helped in reducing the dimensionality and focusing on the most significant features. Afterward, a Rectified Linear Unit (ReLU) function is applied for the model to learn more complex language patterns. The second linear layer maintains the dimensionality at 128 units refining the learnings from the previous layer. The second linear layer was followed by a Tanh (Hyperbolic Tangent) which normalizes the representations into values ranging between −1 and 1. This activation function thus stabilizes the learning process. The final linear layer was pivotal in the regression task as it mapped the 128-dimensional vector to the specific number of regression targets, five personality scores in this case (see Figure 3 for ALBERT model structure customized for regression).

The models were configured with their respective tokenizers and custom regression layers. The tokenizer facilitates the embedding of tokens in a fixed representation in the vector space. These linear layers were instrumental in converting the model outputs into a more suitable form for regression. The architecture was designed to learn and ultimately extract relevant features from textual data to predict personality traits as continuous values. These transformations were aimed at capturing the intricate patterns in the data.

### 3.4 Fine tuning

According to Church et al. (2021), pre-trained models are typically trained on unlabeled datasets for general purposes, whereas fine-tuning calls for the training of the base model on particular downstream tasks with labeled data. Fine-tuning enables us to modify only a few layers of the model's neural network for related but different specialized tasks (Vrbančič and Podgorelec, 2020). In this research design, we used the PyTorch-based versions of RoBERTa and ALBERT with RoBERTa and ALBERT tokenizers for fine-tuning. The training configuration parameters included 40 training epochs, 16 batch sizes, and a maximum token length of 512. The evaluation strategy was set to epochs, and the learning rate was fixed at 2e-5 for both models, with a weight decay of 0.01. These hyperparameters were selected based on a synthesis of previous research and empirical testing. Christian et al. (2021) experimented with 1e-5 and 3e-5 learning rates in addition to 16 and 32 batch sizes in various combinations. We initially used a learning rate of 1e-5 and subsequently increased the value to 2e-5, as supported by Yang et al. (2021) and El-Demerdash et al. (2022). Furthermore, we adopted a batch size of 16 which was substantiated by literature. Increasing the batch size further impeded the training process, due to the available GPU resources requirements. Additionally, El-Demerdash et al. (2022) also recommended setting the token length to a maximum of 512 tokens. A weight decay of 0.01 is generally recommended in pytorch documentation.^3^ Regarding the number of epochs, previous research has utilized a wide range, from three epochs (El-Demerdash et al., 2022) to 60 epochs (Deilami et al., 2022) have been employed. We selected 40 training epochs to sufficiently train the models, simultaneously staying within the designated resource limits. The purpose of this fine-tuning was to optimize the resultant performance of the models while making efficient use of the limited computational resources available. Thus, we initiated the process with values derived from previous works and fine-tuned the hyperparameters as the project progressed. These training arguments were implemented by employing a trainer-class API (Trainer API, 2023) for comprehensive feature training.

### 3.5 Evaluation metrics

The performance of the model was assessed by comparing its predictions with actual values. Evaluation metrics that can discriminate between the method results were used (Deilami et al., 2022). The compute loss functions in RoBERTa and ALBERT were superseded. Functions from Python's Scikit-learn library provide regression metrics for evaluation, including Mean Squared Error (MSE), Mean Absolute Error (MAE), and Root Mean Squared Error (RMSE). The MSE loss is commonly employed as a loss function in regression-based tasks. According to Yang and Huang (2019), a smaller MSE determines the effectiveness of the proposed model.

## 4 Analysis and results

In line with the previous research (Gjurkovic et al., 2021; Yang et al., 2021; Jukić et al., 2022), this study employs the extensive PANDORA dataset, which comprises 27,000 comments from 1,608 authors on the Reddit platform for APP. The dataset was leveraged to train the LLMs, including RoBERTa and ALBERT, with RoBERTa having ~10 times the number of parameters of ALBERT. Tesla P100-PCIE-16GB GPU was used to execute the two model trainings. The predictions entailed a continuous number for each of the Big Five traits, on a scale of 0–100. Furthermore, multi-output regression has been used as a mechanism for the simultaneous prediction of all five traits, as proposed in many studies (Xue et al., 2018; López-Pabón and Orozco-Arroyave, 2022). The execution of text pre-processing, and its input into ALBERT and RoBERTa with the subsequent output regression scores, are illustrated in Figure 2.

**Figure 4:**
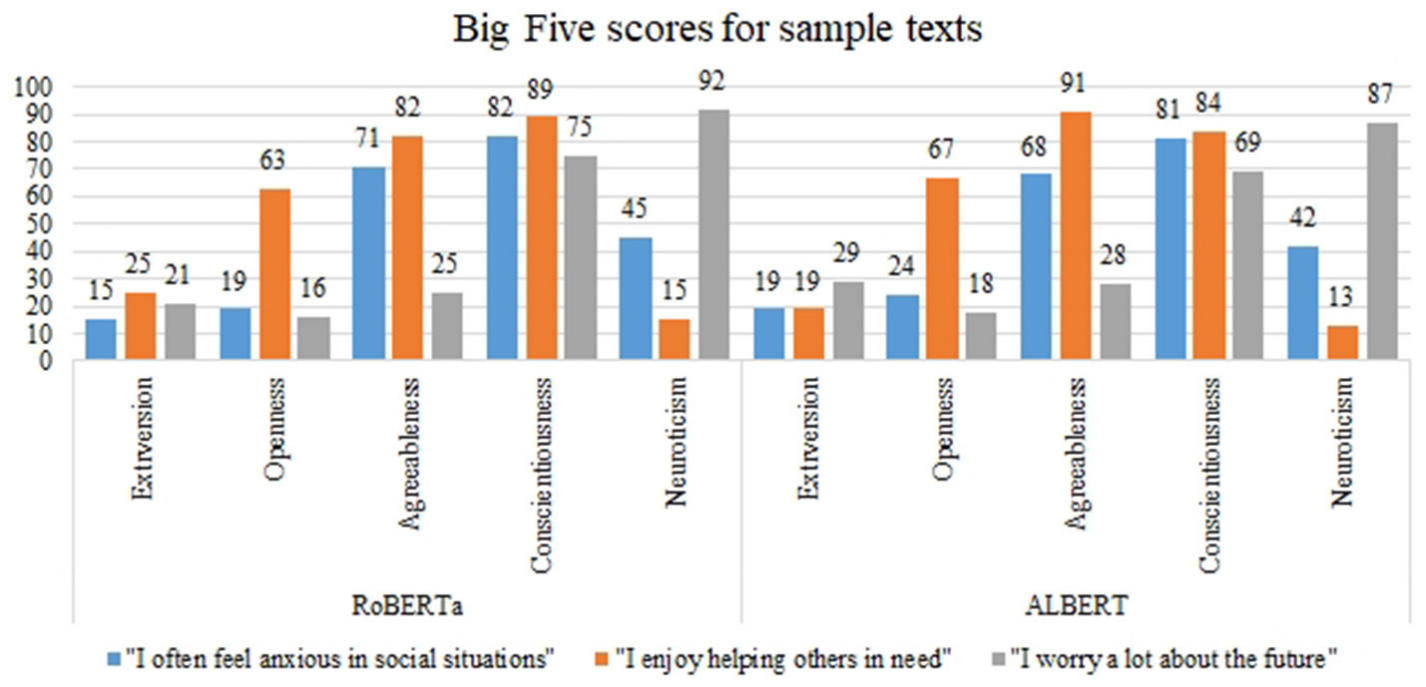
Big Five scores for sample texts.

When evaluating sample texts, both models produced remarkably similar scores on Big Five traits. The predictions made by RoBERTa and ALBERT were consistently close, demonstrating agreement in their assessments. For instance, the input text “I prefer spending time alone with books” yielded comparable predictions with ALBERT scores of “*38, 82, 63, 78, 27”* and RoBERTa scores of “*40, 81, 61, 75, 29”* indicative of extroversion, openness, agreeableness, conscientiousness, and neuroticism respectively. These customized models produce an array of scores for each text input which can be used to comprehend its personality. Figure 4 shows a comparison of the scores predicted by ALBERT and RoBERTa for the given sample texts across all the traits of the Big Five Model, showcasing very close results between the two models. Additionally, Table 6 demonstrates the training loss and reduction in MSE, RMSE, and MAE over 40 epochs while training RoBERTA and ALBERT on the PANDORA dataset. The aforementioned hyperparameters, including the number of epochs, evaluation strategy, learning rate, and batch size of the input, were kept constant for training both models. Despite the differences in their sizes, both models seem to produce similar results in terms of training loss and reduction in MSE, RMSE, and MAE.

**Table 6:** Comparison of evaluation metrics between RoBERTa and ALBERT.

**Epoch**	**RoBERTa**					**ALBERT**				
	**Training loss**	**Validation loss**	**MSE**	**RMSE**	**MAE**	**Training loss**	**Validation loss**	**MSE**	**RMSE**	**MAE**
1	2,508.5298	2,465.8377	2,465.8364	48.9216	39.77474	2,499.2832	2,456.2976	2,456.3022	48.8059	39.6621
5	1,837.4262	1,811.1443	1,811.1420	41.9133	33.25334	1,831.1945	1,804.6882	1,804.6829	41.8195	33.1853
10	1,328.2593	1,305.9076	1,305.9078	35.7156	29.17931	1,325.3960	1,302.8401	1,302.8418	35.6608	29.1646
15	1,045.4495	1,041.1628	1,041.1605	32.0867	27.14586	1,045.0584	1,040.5450	1,040.5442	32.0710	27.1474
20	914.5754	915.7201	915.7194	30.2150	25.95275	915.2727	916.2628	916.2623	30.2219	25.9626
25	848.7351	856.8837	856.8833	29.2569	25.27504	849.6119	857.6313	857.6346	29.2697	25.2848
30	818.1030	828.8650	828.8630	28.7618	24.91060	818.8715	829.5237	829.5251	28.7741	24.9214
35	799.1436	816.3414	816.3398	28.5274	24.71394	799.7687	816.9009	816.9002	28.5383	24.7246
40	800.2508	812.7495	812.7492	28.4584	24.64961	800.8514	813.2720	813.2730	28.4687	24.6602

**Figure 5:**
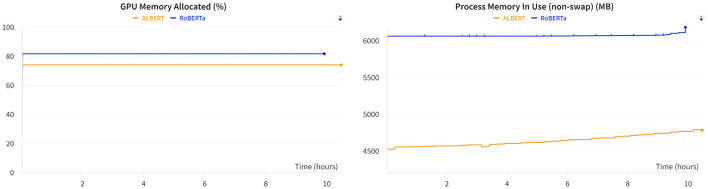
GPU memory consumption during training.

**Figure 6:**
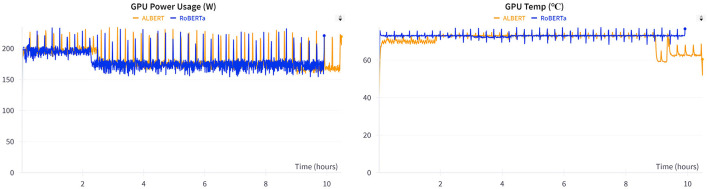
GPU power consumption and heat emission.

Table 7 presents a comparison of the overall performance of the models across 40 epochs. During training, ALBERT consumed 30 min more than RoBERTa because of the slower training steps in ALBERT. However, no significant difference was observed in the overall training loss between the two models. The training loss comparison shows a negligible variance of 0.11%, which is statistically insignificant. Additionally, the metrics in Figure 5 illustrate the GPU memory allocation and resource consumption by RoBERTa and ALBERT when trained separately. ALBERT was found to have a 6% lower allocation of GPU memory, with an average difference of 1,500 MBs overall. Furthermore, Figure 6 illustrates that ALBERT results in a relatively lower emission of heat in GPUs, with differences ranging from 4°C at the beginning, consistent 1°C in the middle, to a significant 14% at the end of the training procedures. Moreover, ALBERT also exhibits relatively lower power consumption in Watts.

**Table 7:** Comparison of training hyperparameters in RoBERTa and ALBERT.

**Model**	**RoBERTa**	**ALBERT**
**Train output**		
Global step	40,440	40,440
Training loss	1,141.85	1,140.59
**Metrics**		
Train runtime	35,939.5414 (9:54:11)	37,620.0265 (10:25:41)
Train samples per second	17.996	17.192
Train steps per second	1.125	1.075
Total flos	0.0	0.0
Train loss	1,141.85	1,140.59
Epoch	40.0	40.0

Taken together, it is interesting to note the striking similarity between the results produced by training both language models. Despite the difference in architecture and number of parameters, training on the same data has led to very close training results. In summary, this study indicates that the size of the language model does not have a discernible difference in learning and the consequent predictions of the model.

## 5 Discussion

This research uncovered two salient aspects of comparison drawn between large and small language models, owing to their parameters. Building upon the notion of textual analysis and its potential to predict the personality of individuals, our study focused on two primary aspects of automated text-based predictions. The initial objective of this study was to explore the feasibility of smaller language models in contrast with LLMs. The second point of focus was the use of multi-output regression to show human personality on a continuum. The resulting values ranged from 0 to 100, exhibiting values for all five traits of the Big Five model employing a large-scale personality dataset. Addressing the aforementioned objectives, the current investigation revealed that no noticeable difference could be observed by training language models on the same dataset. This finding is in line with previous data science research which implies that the quality of the training dataset is crucial in determining the performance of various AI-based models (Stuart Geiger et al., 2020; Liu et al., 2023). Specifically, it reinforces the findings by Mehta et al. (2020b), where they point out that deep learning-based personality prediction is also affected by data quality.

### 5.1 Implications of the study

The contextual alignment of personality detection and computational developments underscore the significance of our study, offering valuable insights building upon the evolving landscape of research in psychology, particularly within the context of APP. First, we shed light on personality predictions using automated methods. Moreover, our research design has been accentuated to incorporate the most recent technological breakthroughs in LLMs, especially transformers proposed by Vaswani et al. (2017). Although pre-trained LLMs have been a less researched methodology, they have become much more desirable areas in research. This desire stems from their sophisticated and resource-intensive computation, with a fraction of the effort and cost invested (Kumar and Renuka, 2023). As mentioned in the literature, transfer learning has revolutionized the realm of NLP (El-Demerdash et al., 2021; Rajapaksha et al., 2021; Yuan et al., 2023) and our empirical investigation reinforces the significance of transfer learning. We found that we can leverage small language models, emphasizing their learning on a specialized task of predicting personality from the textual data, as suggested by recent studies (Araci, 2019; Li et al., 2020; Yang et al., 2020; Kjell et al., 2023). In this study, the execution parameters were kept identical to rule out any other cause of similar results being produced. The findings of this study, presented in Tables 5, 6, show that there is little to no difference in the performance of the training models of varying sizes. This is consistent with the arguments put forth by Hsieh et al. (2023) and Sanh et al. (2019), and supports the idea of smaller language models proposed by Schick and Schütze (2020), after the continuous up-scaling of the language models beyond resources.

Second, in addition to investigating large and small pre-trained LLMs, this study examined online textual data to predict personality, specifically the Big Five traits (Gjurković and Šnajder, 2018). Our approach aligns with the perception of human personality to be evaluated on a continuum instead of labeled classes (Johnson and Murty, 2023). This model investigation follows the work of López-Pabón and Orozco-Arroyave (2022) and Xue et al. (2018), who used regression techniques for APP. We adapted these studies by customizing the pre-trained language models RoBERTa and ALBERT to produce a multi-output regression. This study confirms regression as a viable statistical technique to predict the values of five personality traits, supporting the proposal of Mehta et al. (2020a).

This study offers various practical implications in diverse contexts. APP can be extremely beneficial for maintaining general wellbeing (Moreno et al., 2021) and detecting suicidal tendencies and mental health risks (Deilami et al., 2022). In addition, this concept is expected to be valuable in social network analysis and deception detection (Xue et al., 2018) and voter inclination toward elections (Tutaysalgir et al., 2019). These models can also be deployed to enrich the experience with autocars, robots, voice assistants, and other human-machine interaction agents (Kazameini et al., 2020). Additionally, personality-based psychometric analysis can massively contribute to improvements in crucial business performance indicators such as sales and social media clicks (Matz et al., 2017). Such psychological profiling can influence the behavior of people by personalizing business strategies according to their personalities.

Moreover, our study has utilized a Tesla P100-PCIE-16GB GPU to increase the pace of models' training. The resource consumption statistics of our method validate the feasibility of faster processing units, such as GPUs and TPUs in commercial use. Such advanced processing units together with big data, have enabled companies to adopt state-of-the-art computational methods seamlessly (Lecun et al., 2015). Search engines, recommendation systems, search rankings, fake news identification, and translations are just a few applications already employed in organizations (Pais et al., 2022). Corporations have shown an immensely growing tilt to apply these studies to their business processes.

Since employing models is a challenging task in finding solutions to NLP-related business problems (Paleyes et al., 2022), our research empirically investigated the feasibility of using pre-trained language models to predict the personality traits of individuals from their texts. Furthermore, our research suggests that smaller models can be effectively utilized in diverse business contexts. Additionally, the reduced usage of computational resources lowers the CO_2_e emissions, thereby lowering potential climate impacts, hence addressing the concerns put forward in recent research (Henderson et al., 2020; Patterson et al., 2021). A lower heat emission confirms the decreased necessity for water to cool down the systems, thereby lowering the water footprint reinforcing the argument by Li et al. (2023). According to McDonald et al. (2022), model inference requires nearly 80% of the computational demand. Therefore, analyzing and comparing the computational resource consumption of different LLMs at the training stage is essential to minimize energy consumption and carbon footprint during inference. Our study not only validates the use of pre-trained language models to predict personality but also emphasizes the practicality of employing smaller language models in various organizational settings. Our findings support the previous literature which emphasizes prioritizing energy and computational efficiency when selecting models (Strubell et al., 2020; Tamburrini, 2022). This underscores the practical application of the proposed pre-trained smaller language models in contexts where human personality plays a crucial role. Overall, companies can leverage the benefits of such pre-trained models while minimizing their financial and technical computation budgets, aligning with sustainable business practices.

## 6 Limitations and future implications

We acknowledge the presence of certain limitations in our research. Our research offers initial evidence of the similar performances of a large and a ten times smaller model with other stable parameters. However, because of the unavailability of more powerful GPU resources, we could not include larger models. Models such as Llama (Touvron et al., 2023), LaMDA (Thoppilan et al., 2022), and GPT-3 (Brown et al., 2020) may provide more insightful results as they are much larger. A comparison with such substantial models would offer a more robust perspective on the study. Second, future research could also use datasets with varying sizes in parameters; and quality such as with biased sampling; subjective labels; imbalanced classes; or limited diversity. This would enable a deeper comparison of the models in analyzing which one performs better if data quality is poor.

From the methodological approach, variations in error margin for different texts were observed. Although the model successfully produced the multioutput ratings of the big five traits, the predictions are expected to improve with longer training employing more efficient computational resources. Furthermore, we also propose to contrast the performance output of the auto-encoder and auto-regressive model architectures (Yang et al., 2019; Zhang et al., 2020). This comparison can indicate an architecture that is more suitable for a specialized task of personality prediction. Another architectural comparison can be of single-label classification, multi-label or multi-class classification as well as single-output regression and multi-output regression. Such multi-level analysis may provide insight into the customization criteria for pre-trained models for optimal performance, regardless of their parameter size. Since our study has major implications regarding the use of computational resources by LLMs and its environmental impact, future studies could extend this line of inquiry by employing techniques for carbon-footprint reduction such as power-capping or energy-aware scheduling (McDonald et al., 2022).

## 7 Conclusion

The present study integrates the theoretical underpinnings of the Big Five personality model with state-of-the-art technology. This integration is intended to assess the potential of employing pre-trained language models to predict human personality based on their language. This paper commences with a comparative account of ML and deep learning techniques used for similar objectives by previous researchers. Additionally, our paper highlights the advancements in pre-trained models since their emergence in NLP. Furthermore, our analytical outcomes establish a comparable performance yielded by the two models, RoBERTa and ALBERT, despite their different parameter sizes. Our results also provide logical evidence in support of multi-output regression. Moreover, we observe a reduced heat emission as well as lower carbon and water footprint by smaller models. Our novel findings are expected to stimulate more nuanced questions, to be raised in this direction, thereby broadening the scope of research and industrial applications alike.

## Data Availability

The original contributions presented in the study are included in the article/supplementary material, further inquiries can be directed to the corresponding author.

## Footnotes

^1^The model training is tracked using the platform *Weights and Biases*. This model workflow can be accessed at https://wandb.ai/nlp-thesis/huggingface/workspace?workspace=user-fatima-habib.

^2^The source code for preprocessing text data and model training is available at: https://github.com/Fatima0923/NLP.

^3^ https://pytorch.org/docs/stable/optim.html#torch.optim.AdamW
